# A new mouse model of ADHD for medication development

**DOI:** 10.1038/srep39472

**Published:** 2016-12-20

**Authors:** Petra Majdak, John R. Ossyra, Jessica M. Ossyra, Adam J. Cobert, Gabrielle C. Hofmann, Stephen Tse, Brent Panozzo, Elizabeth L. Grogan, Anastassia Sorokina, Justin S. Rhodes

**Affiliations:** 1 The Neuroscience Program, University of Illinois, IL, USA; 2 The Beckman Institute for Advanced Science and Technology, University of Illinois, IL, USA; 3 Oak Ridge National Laboratory, University of Tennessee, Knoxville, TN, USA; 4 College of Engineering, University of Tennessee, Knoxville, TN, USA; 5 Department of Food Science and Technology, University of California, Davis, CA, USA; 6 College of Veterinary Medicine, University of Illinois, IL, USA; 7 Department of Psychology, University of Illinois, IL, USA.

## Abstract

ADHD is a major societal problem with increasing incidence and a stagnant track record for treatment advances. A lack of appropriate animal models has partly contributed to the incremental advance of this field. Hence, our goal was to generate a novel mouse model that could be useful for ADHD medication development. We reasoned that hyperactivity is a core feature of ADHD that could easily be bred into a population, but to what extent other hallmark features of ADHD would appear as correlated responses was unknown. Hence, starting from a heterogeneous population, we applied within-family selection over 16 generations to produce a High-Active line, while simultaneously maintaining an unselected line to serve as the Control. We discovered that the High-Active line demonstrated motor impulsivity in two different versions of the Go/No-go test, which was ameliorated with a low dose of amphetamine, and further displayed hypoactivation of the prefrontal cortex and dysregulated cerebellar vermal activation as indexed by c-Fos immunohistochemical staining. We conclude that the High-Active line represents a valid model for the Hyperactive-Impulsive subtype of ADHD and therefore may be used in future studies to advance our understanding of the etiology of ADHD and screen novel compounds for its treatment.

Despite the prevalence of Attention-Deficit/Hyperactivity Disorder (ADHD) in our society, and our readiness to dispense pharmaceutical interventions, much of the underlying etiology remains unknown. Core deficits include hyperactivity, inattention, and impaired action inhibition^1^,^2^,^3^. U.S. prevalence rates among children and adults are substantial, and while many children in the U.S. (4.8%) are currently medicated for ADHD, little is known regarding the long-lasting impact of these medications on cognitive health in adulthood^4^,^5^,^6^.

ADHD is a multifactorial, highly heritable disorder, with twin studies estimating a mean heritability of 76 percent^7^. Despite high heritability estimates, the specific network of genes implicated in ADHD are not known^8^. Candidate gene studies that place narrow focus on evaluating components of dopamine and norepinephrine signaling^9^,^10^ are associated with small odds ratios that some have argued may be false positives^11^. Overall, the genes currently associated with ADHD are estimated to account for a small fraction of the total genetic variation^8^,^12^,^13^. A novel approach is needed for a more complete understanding of the genetic underpinnings of ADHD. An improved understanding of the genetic basis of ADHD will lead to improved, targeted therapeutic approaches.

Several animal models of ADHD currently exist but none of them were developed specifically for the purpose of modeling the multifactorial, genetic foundation of ADHD. Single-gene mutant rodent models of ADHD are critical for understanding the contribution of monoaminergic pathways on ADHD pathology and treatment response^14^, yet they are unable to elucidate the broader network of genes mediating behavioral deficits associated with ADHD. Lesion-based models are limited since ADHD is known to involve dysregulation of multiple brain regions, such as the prefrontal cortex (PFC), striatum, and cerebellum^15^,^16^. Arguably the most well studied model of ADHD is the spontaneously hypertensive rat (SHR), which has shown hyperactivity, impulsivity, and inattention^17^. However, the SHR is limited in two important aspects: 1) the model was selectively bred for hypertension, therefore it is difficult to disassociate the effects of hypertension from hyperactivity, and 2) the SHR lacks an appropriate control strain to statistically determine whether phenotypic differences between the lines are related to hyperactivity or other factors. The commonly utilized control, the Wistar-Kyoto rat (WKR), often demonstrates activity levels below that of other rats, and has even been suggested as a model of depression^18^,^19^,^20^,^21^. Therefore, as most studies compare the SHR against only the WKR, it is difficult to determine whether the differences between the strains are related to hyperactivity/ADHD-related phenotypes, hypertension, depression, or any number of other features that differs between these two highly divergent strains.

In the present work, we developed a line of mice specifically to model core features of ADHD along with an appropriate Control line for discovery-based research. Starting from the genetically variable Collaborative Cross population^22^, we have been maintaining 2 lines of mice, one that is bred for increased physical activity in their home cage each generation and the other that is randomly bred with respect to physical activity. We previously reported strong response to selection, correlated responses with other measures of physical activity and paradoxical locomotor responses to amphetamine^23^. Hallmark features of ADHD also include impulsivity and inattention. Further, evidence suggests that AHDH in humans is associated with a hypofunctioning prefrontal cortex and dysregulated cerebellar vermal functioning^16^,^24^,^25^. Hence, the goal of this study was to determine the extent to which the High-Active line recapitulates other core features of ADHD, including motor impulsivity across 2 versions of the Go/No-go task and alleviation of impulsive action via amphetamine administration. A low, therapeutic dose of amphetamine (0.25 mg/kg) that has previously been shown to attenuate hyperactivity in the High-Active line while exacerbating activity in the Control line^23^, was selected for evaluating its potential efficacy and relevance for ameliorating motor impulsivity. Finally, High-Active mice were evaluated for inattention on the Y-maze, and the functionality of implicated brain regions such as the PFC and cerebellum.

## Materials and Methods

### Animals

Mice from Generation 15 (Experiment 1) and Generation 16 (Experiments 2 & 3) of a selective breeding experiment for increased distance traveled in the home cage were used^23^,^26^. Our lab maintains two lines of genetically variable mice: a randomly bred, unselected (Control) line and a line selectively bred for increased distance traveled in the home cage (High-Active). Inbreeding is minimized via within-family selection. The starting population for each line was generated by systematically crossing 8 different inbred strains, chosen to maximize genetic variation (Collaborative Cross mice^22^). Each generation at weaning on postnatal (PND) 21, mice are group-housed by sex. At approximately PND 60, mice are phenotyped for home cage activity. Mice are placed in custom-made acrylic home cages (18.5 cm × 33.5 cm × 16 cm) with clear plastic lids that allow continuous ceiling-mounted video tracking by TopScan (CleverSystems, Reston, VA, USA). Each cage individually houses 4 mice by sex, with an interaction zone constructed of wire mesh that allows for limited physical contact and interaction. The video coverage allows for continuous tracking of 64 individual mice over the span of 6 days. Well after an extended habituation period of 4 days, the average distance traveled during days 5 and 6 is used as the selection criterion^23^,^26^.

### General Husbandry

Rooms are kept controlled for temperature (21 ± 1 °C) and photo-period (12:12 L:D; lights on at 8:00 PM and off at 8:00 AM). All behavioral testing occurred during the dark cycle. Food (Harlan Teklad, 7012) and water were provided ad libitum, except during operant training and testing. Mice were group housed by sex after weaning, individually phenotyped in custom-built plexiglass cages, and then individually housed in standard shoebox cages. Corncob bedding (7097 Harlan Teklad, Madison, Wisconsin, USA) was provided in all cages. The Beckman Institute Animal Facility is AAALAC approved. All procedures were approved by the University of Illinois Institutional Animal Care and Use Committee and adhered to NIH guidelines.

### Apparatus for Operant Conditioning

For Go/No-Go testing, 6 identical modular operant test chambers (12 × 9.5 × 8.25 in) for mice (Med Associates Inc., St. Albans, VT) were each housed within a standard MDF sound-attenuating cabinet (22 × 15 × 16 in). A house light (28 V DC, 100 mA) is installed opposite the nose poke holes. A tone generator (Sonalert, 2,900 Hz) is mounted next to the house light. Within the chamber are three nose-poke recesses. The left and right recesses have a yellow LED stimulus (cue light) directly within the hole. The center nose-poke recess has no light but is equipped with a lever to dispense a sucrose reward (10% w/v) via a 0.01 cc stainless steel cup. The grid floor of the chamber consisted of 24 stainless steel rods (0.13 inch diameter rods spaced at 0.31 inches) mounted in polycarbonate supports. A computer program written in MED-PC (Med Associates Inc.) controlled the output devices and recorded responses.

### General Procedure for Operant Conditioning & Variables of Interest

**Figure 1:**
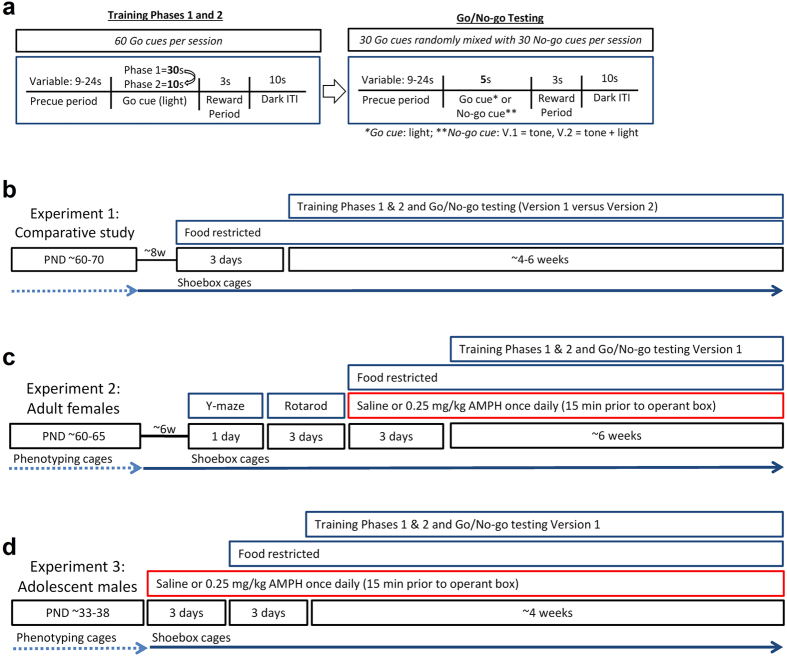
Experimental schematics. (**a**) This schematic represents the context in which all cues are presented. First, the house light is illuminated at the onset of the variable precue period. A correct response (nose poke for Go cue, withholding nose poke for No-go cue) dispenses a small sucrose reward (an incorrect response bypasses the reward period). Lastly, the house light is extinguished for 10 seconds. In Training Phase 1, sixty 30-second Go cues (lights) are presented, but 30 nose pokes in response to the cue light in under 40 minutes for two consecutive days advances the mouse to Training Phase 2, in which the cue light is illuminated for only 10 seconds (reinforcing the prepotent motor response). The same 30 hits/40 minutes criterion advances mice to Go/No-go testing, in which thirty 5-second Go cues are randomly interspersed with thirty 5-second No-go cues. While both versions of Go/No-go testing had the same Go cue (light), in Version 1 (V.1) mice underwent 10 days of testing with a tone No-go cue, while in Version 2 (V.2) mice underwent 20 days of testing with a concurrent tone + light No-go cue. Version 1 was implemented for subsequent Experiments 2 and 3. (**b**) In Experiment 1, disparate Go/No-go methods assessed impulsivity across paradigms. Adult male and female mice were phenotyped and food restricted throughout training and testing. At Go/No-go testing, paradigms diverge between Versions 1 and 2 as outlined in 1a. (**c)** Experiment 2 utilized the most hyperactive cohort of mice (adult females), which were first assessed on the Y-maze and accelerating rotarod, before concurrently acclimating to food restriction and saline or 0.25 mg/kg d-amphetamine i.p injections over 3 days. Food restrictions and injections 15 minutes prior to operant chamber placement continued throughout training and testing. (**d)** Experiment 3 utilized adolescent males that were phenotyped for home cage activity, acclimated to daily i.p. injections of saline or 0.25 mg/kg d-amphetamine over 3 days, and food restricted over an additional 3 days. Mice received injections administered 15 minutes prior to placement in the operant chamber.

We followed the general procedure outlined by Mitchell and colleagues to compare Go/No-go impulsivity measures in multiple strains of mice, as this paradigm provided published heritability estimates^27^. Mice were food restricted for 3 days prior to Training Phase 1, and this food restriction continued throughout operant training and testing. After successfully completing training Phase 1 by achieving 30 correct “hits” (nose poke response to the Go cue light) in under 40 minutes, for two consecutive days, mice progressed to Phase 2. In Phase 2, the cue was illuminated for only 10 seconds. After completing Phase 2 using the same criteria as Phase 1, mice are considered to have an established prepotent motor response^27^ (Fig. 1a). Not all mice passed these training phases, hence resulting in the unbalanced sample sizes listed for each subsequent experiment (full exclusion details provided in Supplemental Materials). Mice then underwent Go/No-go testing as outlined below. While sucrose preference testing was not specifically performed, all mice that failed to collect their earned sucrose reward during Go/No-go testing were excluded from analysis.

Variables of interest from Go/No-go testing included hits (nose pokes in response to a Go cue light), false alarms (incorrect nose pokes in response to a No-go cue), the latency to make a hit or false alarm, precue responses (nose pokes during the last 3 seconds of the precue period), cue-side pokes (random poking in cue recess in absence of any cue, i.e. during the precue, reward, or darkened intertrial interval periods), and efficiency (total reinforcers earned/total number of nose pokes).

### Experiment 1: Evaluating two discrepant Go/No-go procedures

Impulsivity was measured in High-Active and Control mice using two Go/No-go procedures that differed in the sensory cues used to elicit inhibition. In Version 1, testing was conducted over 10 daily sessions during which the Go cue was the nose poke cue light, and the No-go cue was a tone. This version closely resembles the published procedure across multiple strains^27^. In Version 2, Go/No-go testing was conducted over 20 daily sessions during which the Go cue is the nose poke cue light, while the No-go cue is the simultaneous presentation of the Go cue and a tone. This version is similar to previously published methods using concurrent light and tone to signal a No-go cue^28^,^29^, and was included because it demands a stronger level of behavioral inhibition than Version 1. In Version 1 the mouse must inhibit prepotent responding to a tone that was never paired with reward, but in Version 2 the mouse must inhibit their prepotent response to the light cue that was originally paired with reward when it co-occurs with a tone.

A total of 22 High-Active (n = 11 males and n = 11 females) and 16 Control (n = 8 males and n = 8 females) mice were phenotyped for home cage activity at PND 60–70. Individuals were from distinct families within each sex. Eight weeks after home cage phenotyping, mice (PND 116–126) were trained and then tested for Go/No-go behavior using Version 1 or Version 2 described above (Fig. 1b). At the start of training, 19 mice were assigned to Version 1, and 19 to Version 2. However, only the following mice made it through exclusion criteria for training and reward consumption: High-Active mice (Version 1: n = 7 mice [3 males, 4 females]; Version 2: n = 7 mice [4 males, 3 females]) and Control mice (Version 1: n = 7 [4 males, 3 females]; Version 2: n = 7 mice [4 males, 3 females]).

### Experiment 2: Adult females

Adult female High-Active (n = 20) and Control (n = 23) mice were used for this experiment. Individuals were from distinct families within each sex. Mice were phenotyped for home cage activity from PND 60–65. Approximately 6 weeks later, mice were tested on the Y-maze (1 day) and rotarod (3 days). Following rotarod, mice began receiving daily injections of 0.25 mg/kg d-amphetamine or saline concurrent with food restriction, and after 3 days of acclimation to this schedule began training on Version 1 of Go/No-go (Fig. 1c). On days when animals were being trained and tested on the Go/No-go task, injections occurred exactly 15 minutes prior to placement in the operant boxes. Following the Go/No-go testing mice were euthanized for immunohistochemical detection of c-Fos in the infralimic and prelimbic cortices (PFC), and posterior cerebellar vermal lobules.

#### Y-maze

The maze consisted of 3 identical arms (15 × 3 × 5 in) made of black Plexiglas. The top of the maze was not covered, and continuous ceiling-mounted video tracking by TopScan recorded arm entries. At the start of each trial, a mouse was placed in the center of the maze and allowed to freely explore for the entire 8-minute duration. The sequence in which the mouse entered the arms of the maze was recorded manually. An arm entry was defined as the entry of all four paws into the arm. An alternation was defined as consecutive entry into all three arms (without revisiting an arm). The total number of arm entries was also recorded.

#### Rotarod

Mice were placed on the stationary rotarod dowel (AccuRotor Rota Rod Tall Unit, 63 cm fall height, 30 mm diameter rotating dowel; Accuscan, Columbus, OH), which was then accelerated at 60 rpm/min. The latency to fall (in seconds) was recorded. The procedure was repeated for 4 consecutive trials per day. If an animal fell off the rotarod rapidly due to inattention or slips, they were given an additional trial. The entire procedure was repeated for a total of 3 days. Average and maximum latencies across the 4 trials per day were analyzed.

#### Go/No-go

The following mice successfully completed the operant training: High-Active mice (n = 8 receiving saline, and n = 10 receiving 0.25 mg/kg amphetamine) and Controls (n = 4 receiving saline, and n = 9 receiving 0.25 mg/kg amphetamine).

#### c-Fos Immunohistochemistry and Image Analysis

Only mice that successfully completed the operant training were analyzed for c-Fos (see Go/No-go section above). Mice were euthanized exactly 90 minutes following their final injection of either saline or 0.25 mg/kg amphetamine. Briefly, mice were anesthetized with 100 mg/kg sodium pentobarbital via intraperitoneal injection, and then perfused transcardially with ice-cold saline. Brains were immediately dissected. The left hemisphere and intact cerebellum were placed in 4% paraformaldehyde in phosphate buffer solution (PBS) overnight, and then transferred to 30% sucrose solution with sodium azide at 4 °C.

A cryostat was used to section the left hemispheres into 40 μm coronal sections and cerebellum into 40 μm sagittal sections that were then stored in tissue cryoprotectant at −20 °C. Immunohistochemical detection of c-Fos was performed as previous described^30^. Primary c-Fos antibody concentrations were 1:3000 for PFC, and 1:500 for cerebellum (Santa Cruz, sc-52). A 1:6 series was stained for PFC (i.e., series of sections throughout the rostrocaudal extent with 240 μm separating each section) while a 1:5 series (200 μm separating each section) was stained for cerebellar vermis. A bright field microscope 10x objective (total magnification 100X) interfaced to computer via AxioCam camera was used to photograph the prelimbic and infralimbic cortices (from Bregma, anterior + 1.98 mm to + 1.54 mm)^31^. Prelimibic and infralimbic cortical regions were traced using anatomical relationship to the emerging corpus callosum and the total number of c-Fos positive cells within the field was hand counted. For the cerebellum, the granule layer of posterior vermal lobules VI and VII (from Bregma, lateral −0.04 mm to +1.44 mm)^31^ were outlined and c-Fos cells were counted using an automated threshold set in ImageJ.

### Experiment 3: Adolescent males

A total of 20 High-Active and 19 Control male mice were phenotyped for home cage activity from PND 33–38. In total, these mice represented 11 families, and were born within 4 days of each other. As outlined in Fig. 1d, immediately after home cage activity was recorded, mice began receiving daily intraperitoneal injections of 0.25 mg/kg d-amphetamine (Sigma A-5880; Lot # 065K1894) or saline vehicle. Food restriction occurred after 3 days of these injections. Following three days of food restriction, mice started operant training and completed Version 1 of Go/No-go testing. On days when animals were being trained and tested on the Go/No-go task, injections occurred exactly 15 minutes prior to placement in the operant boxes. This schedule was implemented to ensure the mice were experiencing the same degree of psychoactive effects from amphetamine while performing the operant tasks. The injections continued every day throughout the duration of the study up until the day of euthanasia. Only the following mice successfully met criteria: male High-active mice (n = 5 receiving saline, and n = 7 receiving 0.25 mg/kg amphetamine) and Controls (n = 4 receiving saline, and n = 5 receiving 0.25 mg/kg amphetamine).

### Statistical Analysis

Data were analyzed using SAS (version 9.3) statistical software. In all analyses, P ≤ 0.05 was considered to be statistically significant.

All data were tested for normality before using parametric statistical analyses. Specifically, standard residuals were plotted and tested for skewness not to exceed |1| and kurtosis between 2 and 3. In the event that data were not normally distributed, data were power- or log-transformed to meet our criteria for normality.

#### Home cage activity

Home cage distance traveled on days 5 and 6 was compared between High-Active and Control lines using an unpaired t-test for each experiment separately. Only data from individuals that completed the operant training are analyzed and represented. Data were collapsed across sex for Experiment 1 because the sample sizes were too low for sufficient power to detect sex differences.

#### Operant training

Mice were trained to respond (Go) to a cue light by poking their nose in the hole containing a light up to a certain criterion (see *General Procedure for Operant Conditioning*). Average latency to respond on the last 4 days of training up to the point when criterion was reached was analyzed by repeated measures ANOVA with line, version (Experiment 1), amphetamine treatment (Experiments 2 & 3), and day (within subjects) as factors.

#### Go/No-go

Measures of performance across all days of Go/No-go testing (number of hits, false alarms, latency to nose poke, precue pokes, cue-side pokes, and efficiency) were averaged for each mouse, and were analyzed with line and version as factors for Experiment 1, and treatment and line as factors for Experiments 2 and 3. False alarms were also analyzed by analysis of covariance with home cage activity, number of hits, or both entered as covariates, to evaluate the line differences after removing variation explained by these variables. In Experiments 2 and 3, false alarms were analyzed by analaysis of covariance only for mice exposed to saline because amphetamine was predicted to abolish the line differences. Precue responses and cue-side pokes were log transformed to improve homogeneity of variance between groups.

#### Y-maze, rotarod, and c-Fos

In Experiment 2, number of Y-maze arm entries and alternations were analyzed by unpaired t-tests comparing High-Active to Control. Average and maximum latency to fall from the rotarod were analyzed using a repeated-measures ANOVA with line and day (within subjects). The average number of c-Fos positive cells (cerebellar vermis lobules VI and VII data were log transformed to improve homogeneity of variance between groups) were analyzed by two-way ANOVA, with treatment and line as factors. One individual was removed from analysis of all brain regions due to poor staining.

#### Genetic drift

It is critical to assess whether phenotypic differences between High-Active and Control lines are due to selection for hyperactivity or simply due to random factors such as genetic drift or founder effects that inevitably occur when two populations are reproductively isolated from one another over multiple generations. Therefore, variance expected from genetic drift was calculated for each trait following Majdak *et al*.^23^. These tests were performed on all traits for which relevant heritability estimates have been previously established. Briefly, in order to calculate standardized phenotypic differences between the lines (D_y_), Control trait means were subtracted from High-Active trait means, and divided by the pooled estimates of the standard deviation for that line’s trait. This D_y_ value was then compared to the 95% confidence interval for genetic drift, which was estimated using our inbreeding coefficient (“F”) generated by ASReml-R version 2.0 32, heritability (“h^2^”) values obtained from the literature, and the number of families used in the phenotypic measurement (“n”). The absolute values of D_y_ that fall out the confidence interval are likely correlated responses to selection as opposed to genetic drift.

## Results

### Home cage activity

**Figure 2:**
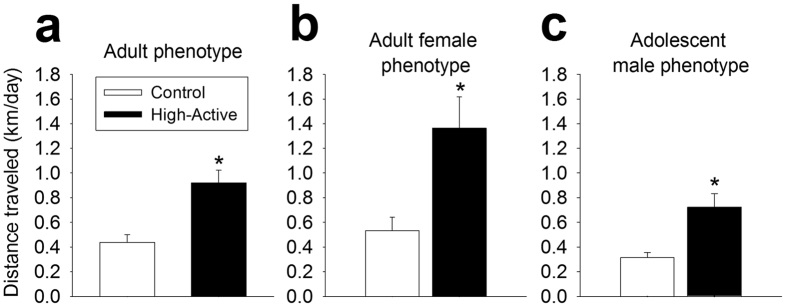
Distance traveled in the home cage setting. Data represent the average distance traveled in the home cage (km/day ± SEM) across days 5 and 6 of a six-day test. These data reflect only those Control and High-Active mice that completed operant training and Go/No-go testing. Asterisks represent statistically significant line differences (P < 0.05) (**a)**. Home cage activity of adult male and female mice used in Experiment 1 (n = 14 Control mice, 8 males and 6 females; n = 14 High-Active mice, 7 males and 7 females). (**b)** Home cage activity of adult female mice used in Experiment 2 (n = 13 Control female mice; n = 18 High-Active female mice). (**c)** Home cage activity of adolescent males used in Experiment 3 (n = 12 Control male mice; n = 9 High-Active male mice).

As expected, High-Active mice were significantly more active than Controls in each experiment (Fig. 2; Experiment 1: t_26_ = 16.7, P = 0.0004; Experiment 2: t_29_ = 7.05, P = 0.013; Experiment 3: t_19_ = 9.8, P = 0.006). Only mice which successfully completed Go/No-go testing and training are represented in these data.

### Training performance

**Figure 3:**
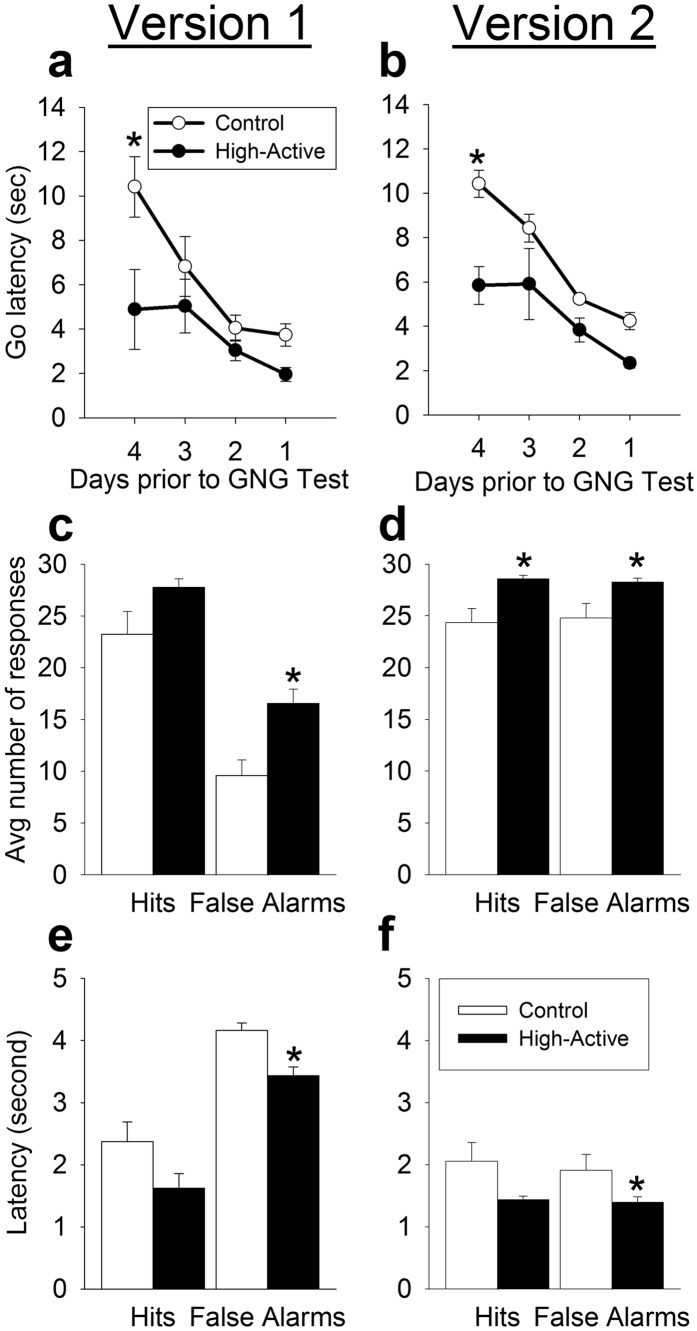
Experiment 1: Evaluating High-Active and Control performance in two discrepant Go/No-go procedures. White bars/circles represent Control mice while dark bars/circles represent High-Active mice. Version 1 means (No-go cue = tone) are shown in the left panels, while Version 2 means (No-go cue = tone + light) are in the right panels. The sample sizes for the data shown are as follows: Version 1 included 7 High-Active mice (3 males, 4 females) and 7 Control mice (4 males, 3 females), and Version 2 included 7 High-Active mice (4 males, 3 females) and 7 Control mice (4 males, 3 females). Asterisks represent statistically significant line differences (P < 0.05). (**a,b)** Data represent the mean latency in seconds (±SEM) for the Version 1 and Version 2 cohorts to respond to the presentation of a Go cue (light) during the last four days of their training before commencing Go/No-go testing. Both versions employed an identical Go cue. (**c**) The average number of hits (correct nose poke in response to a light) and false alarms (incorrect nose pokes in response to a tone) (±SEM) over 10 days of Go/No-go testing in Version 1. A maximum of 30 hits and 30 false alarms is possible. (**d**). The average number of hits (correct nose poke in response to a light) and false alarms (incorrect nose pokes in response to a tone + light) (±SEM) over 20 days of Go/No-go testing in Version 2. A maximum of 30 hits and 30 false alarms is possible. (**e**) The average latency in seconds to make a hit or a false alarm across 10 days of Go/No-go testing in Version 1. A maximum latency of 5 seconds is possible. (**f**) The average latency in seconds to make a hit or a false alarm across 20 days of Go/No-go testing in Version 2. A maximum latency of 5 seconds is possible.

In all three experiments, High-Active mice displayed significantly faster latencies to nose-poke in response to the cue light. However, this difference dissipated toward the end of the training session as the latencies for Control mice dropped to similar levels as High-Active mice, indicating both groups displayed a similar prepotent motor response prior to Go/No-go testing (see Fig. 3a,b). In Experiment 1, this was indicated by significant effect of line (F_1,22_ = 14.3; P = 0.001), significant effect of day (F_3,66_ = 30.99; P < 0.0001), and a significant interaction between day and line (F_3,66_ = 4.78; P = 0.005). As expected, no training difference was detected between Go/No-go Version 1 and Version 2, nor were there any interactions with the other factors, confirming that the mice were similarly trained going into the Go/No-go testing. Post-hoc Tukey tests indicated High-Active mice were significantly faster to respond than Controls on Day 1 (P < 0.0001), but not on the other days. Experiments 2 and 3 followed a similar training pattern, wherein line differences were nonexistent just prior to Go/No-go testing (Experiment 2: day effect F_3,96_ = 51.6, P < 0.0001; Experiment 3: day effect F_3,87_ = 24.2, P < 0.0001 and day by line interaction F_3,87_ = 4.61, P = 0.005).

### Experiment 1: Evaluating two discrepent Go/No-go procedures

#### Hits and false alarms

Out of 30 possible hits during a Go/No-go session, High-Active mice on average, demonstrated significantly more hits than Controls (F_1,18_ = 9.2; P = 0.007; collapsed hits across versions for Fig. 3c,d). No effect of Go/No-go version was detected. In addition, High-Active mice made significantly more false alarm errors than Controls in both versions of the task (F_1,18_ = 18.0; P = 0.0005; Fig. 3c,d). As expected, number of false alarms was much greater for Version 2 than Version 1 of the Go/No-go test (F_1,18_ = 115.9; P < 0.0001), indicating mice had difficulty acquiring the meaning of the No-go cue in Version 2. The increased number of hits and false alarms in the High-Active line relative to the Control line was significantly greater than would be expected from genetic drift alone (Table 1), indicating that selection for hyperactivity likely causes the differences in operant responding between High-Active and Control lines, as opposed to confounds related to reproductive isolation over multiple generations.

#### Analysis of Covariance of False Alarms

In Version 1, number of hits was a significant predictor of number of false alarms (F_1,11_ = 25.6; P = 0.0004), and after including number of hits as a covariate, line remained significant (F_1,11_ = 5.8; P = 0.035), with the High-Active line displaying approximately 4 more false alarms for a given number of hits. This indicates impulsive action in the High-Active line is not a trivial consequence of their increased number of hits. Home cage activity was also a significant predictor of number of false alarms in Version 1 (F_1,11_ = 12.0; P = 0.005), and after correcting for cage activity, the line difference was no longer significant. In Version 2, hits (F_1,9_ = 198.1; P < 0.0001) and home cage activity (F_1,9_ = 7.3; P = 0.02) were significant predictors of number of false alarms, and including these as covariates either together or alone washed out the significance of the line differences. In every case, the pattern was for High-Active to display more false alarms than Controls for a given number of hits or distance traveled in the home cage.

#### Latency to nose poke

High-Active mice responded significantly more quickly to the Go cue than Controls (F_1,18_ = 6.7; P = 0.019; collapsed hits across versions for Fig. 3e,f). No difference in version or interaction between version and line was detected. In addition, High-Active mice mistakenly responded significantly more quickly to the No-go tone cue than Controls (F_1,18_ = 10.9; P = 0.004; Fig. 3e,f). As expected, the latency to make an error and respond to the No-go cue was much longer for Version 1 than 2, since Version 2 was a more difficult task (F_1,18_ = 10.9; P = 0.004; Fig. 3f). Decreased latency to commit a false alarm is likely a correlated response to selection for increased home cage activity rather than genetic drift for both versions, while latency to perform a hit did not survive the test for drift (Table 1).

### Experiment 2: Adult females

Adult females were used for this experiment because they display exaggerated levels of home cage hyperactivity relative to males, not only within our High-Active and Control lines, but in all strains of mice of which we are aware^30^. If enhanced hyperactivity correlates with worsened motor impulsivity then we hypothesize that this adult female cohort would be best suited to demonstrate highly impulsive/inattentive behavior.

#### Y-maze

**Figure 4:**
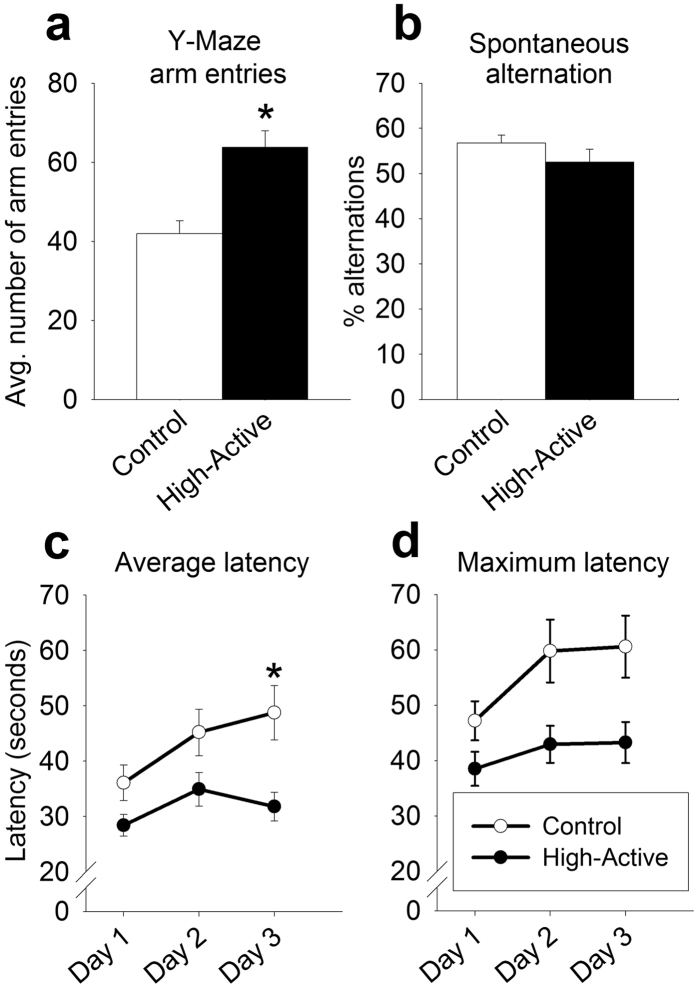
Experiment 2: Adult female performance in the Y-maze and accelerating rotarod. White bars/circles represent Control mice while dark bars/circles represent High-Active mice. The sample sizes for the data shown are as follows: for the High-Active cohort, n = 20; for the Control cohort, n = 23. Asterisks represent statistically significant line differences (P < 0.05). (**a)** The average number of arm entries (±SEM) made during an 8 minute Y-maze free exploration by adult female High-Active and Control mice. (**b)** The average percentage of spontaneous alternation behavior (±SEM) made by adult female High-Active and Control mice across the entire 8 minute trial. A spontaneous alternation was defined as consecutive entries into all three arms without revisiting an arm. (**c)** The average latency in seconds (±SEM) to fall off an accelerating rotarod dowel. Each mouse performed 4 consecutive trials per day, which were averaged together, across 3 days. (**d)** The maximum latency in seconds (±SEM) to fall off an accelerating rotarod dowel. The best trial (i.e., longest latency to fall) of the 4 daily trials completed by each mouse was used to generate averages across the 3 days.

Successful nagivation of the Y-maze is measured by spontaneous alternation behavior, as it requires aspects of attention and working memory^33^,^34^. We therefore hypothesize this behavior may be impaired in the High-Active model of ADHD. High-Active mice displayed significantly more arm entries than Control mice throughout the duration of the Y-maze (F_1, 41_ = 18.12, P = 0.0001; Fig. 4a). Specifically, High-Active mice averaged 64 arm entries while Control mice averaged 42 entries. When all arm entries during the entire eight minute trials were analyzed, the percentage of alternation was similar between the High-Active (53%) and Control mice (57%) (F_1, 41_ = 1.75, P = 0.193; Fig. 4b). The difference in number of arm entries between High-Active and Control mice likely arose as a correlated response to selection for hyperactivity (Table 1).

#### Rotarod

Rotarod is considered an index of cerebellar function^35^, and as cerebellar functioning is hypothesized to be dysfunctional in individuals with ADHD^16^ this task serves as a proof-of-concept that cerebellar activity is impaired in the High-Active line and warrants further investigation. All mice learned the rotarod task as indicated by a main effect of day for both average latency to fall (F_2, 82_ = 12.45, P < 0.0001; Fig. 4c) and maximum latency (F_2, 82_ = 10.54, P < 0.0001; Fig. 4d). However, High-Active mice performed significantly worse than Controls. This was indicated by a significant main effect of line (average latency, F_1, 41_ = 6.40, P = 0.015; maximum latency F_1, 41_ = 6.24, P = 0.017), and interaction between line and day for average latency (F_2, 82_ = 3.41, P = 0.038) but not maximum latency. Post-hoc tests indicate lines differ on day 3 for average latency (p = 0.015). The difference in average latency to fall from the rotarod (across all 3 days) between High-Active and Control mice did not survive the test for genetic drift (Table 1).

#### Hits and False Alarms

**Figure 5:**
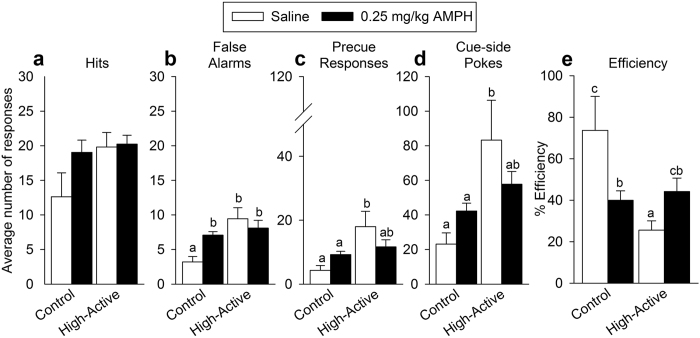
Experiment 2: Adult female response to low-dose amphetamine in Go/No-go. White bars indicate saline-exposed mice while dark bars represent 0.25 mg/kg amphetamine-exposed mice. Intraperitoneal injections were administered 15 minutes before performing Go/No-go testing. Bars with different letters are significantly different from each other. The sample sizes for the data shown are as follows: for the High-Active cohort, n = 8 received saline and n = 10 received 0.25 mg/kg amphetamine; for the Control cohort, n = 4 received saline and n = 9 received 0.25 mg/kg amphetamine. All data reflect the 10 day average across Go/No-go testing. (**a**) The average number of hits (correct nose poke in response to a Go cue light) (±SEM) performed by High-Active and Control mice across Go/No-go testing. (**b**). The average number of false alarms (incorrect nose pokes in response to a No-go tone cue) (±SEM) performed by High-Active and Control mice across Go/No-go testing. (**c**) The average number of precue responses (±SEM), defined as nose pokes made in the 3 seconds prior to the cue presentation, across Go/No-go testing. (**d**) The average number of nose pokes (±SEM) made in the nose poke recess that contains the cue light, in the absence of any cue presentation, across Go/No-go testing. (**e**) The average percent efficiency (total number of reinforcers obtained for correct responding/total number of nose pokes made x 100), across Go/No-go testing.

Significantly more hits were made by High-Active mice in response to a Go cue (F_1, 27_ = 4.24; P = 0.049), but amphetamine had no significant effect in either line (Fig. 5a). More false alarms were committed by High-Active mice (F_1, 27_ = 8.64; P = 0.007), and while there was no main effect of amphetamine, there was a significant interaction between line and amphetamine (F_1, 27_ = 4.46; P = 0.044; Fig. 5b). This is because low-dose amphetamine increased the number of false alarms in Control mice (F_1, 17_ = 16.61; P = 0.001) without affecting High-Active mice. The high number of false alarms committed by High-Active mice is likely due to selection for home cage hyperactivity (Table 1).

#### Analysis of Covariance of False Alarms

Considering only mice exposed to saline, number of hits was not a significant predictor of false alarms. After including hits as a covariate, the difference between the lines was no longer significant. Home cage activity was not a statistically significant predictor of number of false alarms, but after including it as a covariate, High-Active mice performed approximately 9 more false alarms than Controls (F_1,9_ = 12.4; P = 0.007). In the model that included both hits and home cage activity as covariates, High-Active mice displayed approximately 7 more false alarms than Controls (F_1,8_ = 10.5; P = 0.01). In addition both the covariates reached statistical significance (hits, F_1,8_ = 7.2; P = 0.03; cage activity, F_1,8_ = 7.8; P = 0.02). These results indicate that the increased number of false alarms observed in the High-Active adult females is not a trivial consequence of their increased number of hits or hyperactivity.

#### Additional measures of operant responding

Impulsive precue responding, defined as nose pokes within the final 3 seconds of the precue period, was significantly increased in High-Active mice (F_1, 27_ = 11.88; P = 0.002), and while there was no main effect of amphetamine, there was a significant interaction between line and amphetamine (F_1, 27_ = 8.73; P = 0.006; Fig. 5c). This is because low-dose amphetamine tended to reduce precue responses (i.e., enhanced impulse restraint) in High-Active mice whereas it had no significant effect in Control mice. Moreover, High-Active mice made significantly more nose pokes on the cue-side hole while it was not illuminated (F_1, 27_ = 14.33; P = 0.0008), and while there was no main effect of amphetamine, there was a significant interaction between line and amphetamine (F_1, 27_ = 5.85; P = 0.023; Fig. 5d). The interaction was caused by amphetamine reducing cue-side pokes in High-Active mice while increasing it in Controls; these data reflect the capacity of amphetamine to reduce random prepotent responding in hyperactive mice. The efficiency measure (providing minimal responses for maximal reward) indicated that High-Active mice were responding less efficiently for rewards (F_1, 27_ = 9.19; P = 0.005), and while there was no effect of amphetamine, there was a significant interaction between line and amphetamine (F_1, 27_ = 13.05; P = 0.001) (Fig. 5e). Amphetamine reduced efficiency in Control mice (P = 0.006) whereas it increased efficiency in High-Active mice (P = 0.05).

#### Immunohistochemical Analysis

**Figure 6:**
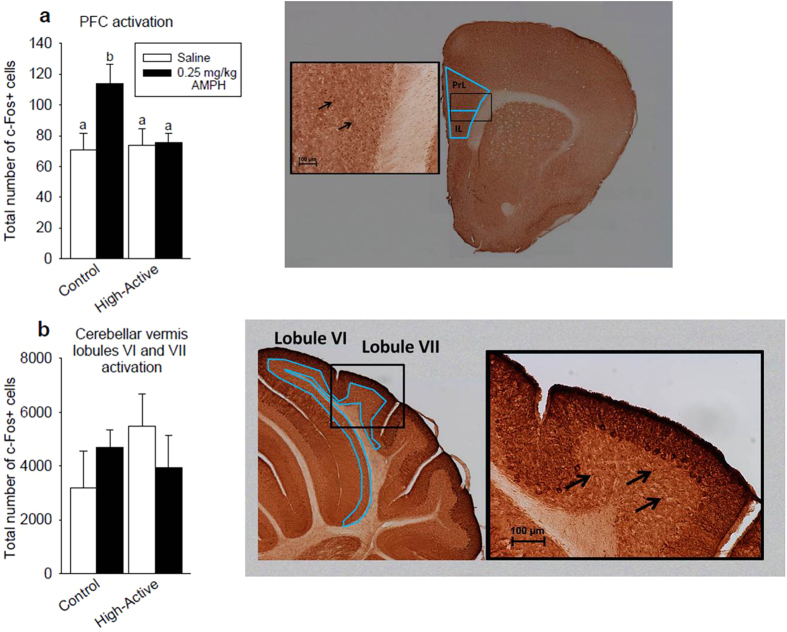
Experiment 2: Adult female response to low-dose amphetamine in regional neural activation. White bars indicate saline-exposed mice while dark bars represent 0.25 mg/kg amphetamine-exposed mice. Bars with a different letters are significantly different from each other. Intraperitoneal injections were administered 90 minutes before sacrifice in order to obtain sufficient c-Fos activation for immunohistochemical analyses. Representative images are shown. Light blue tracings denote regions of interest outlined, and arrows indicate c-Fos punctae. The sample sizes for the data shown are as follows: for the High-Active cohort, n = 8 received saline and n = 10 received 0.25 mg/kg amphetamine; for the Control cohort, n = 4 received saline and n = 9 received 0.25 mg/kg amphetamine. (**a**) The total number of c-Fos positive (activated) cells in the prefrontal cortex, i.e. the combined prelimbic (PrL) and infralimbic (IL) cortices, of High-Active and Control mice exposed to either saline or 0.25 mg/kg d-amphetamine. (**b**) The total number of c-Fos positive (activated) cells in the granular cell layer of cerebellar vermis lobules VI and VII of High-Active and Control mice exposed to either saline or 0.25 mg/kg d-amphetamine.

The purpose of the immunohistochemical analysis was to test our hypothesis that hypoactivation of regions implicated in ADHD would be detected in High-Active mice, specifically within the PFC (pre- and infra-limbic cortices) and the posterior cerebellar vermis, at baseline (saline). Such deficits may be ameloriated by low-dose (0.25 mg/kg) amphetamine. These regions were analyzed to provide construct validity for the High-Active line, as the PFC and cerebellum are influenced by amphetamine and have also been shown to be correlated with ADHD-like behavior^2^,^16^. High-Active mice displayed reduced activation of the infralimbic and prelimbic cortices as compared to Control mice (F_1, 31_ = 6.42; P = 0.017). The main effect of amphetamine and interaction between line and amphetamine did not reach statistical significance. Posthoc tests indicated that the Control line given amphetamine displayed approximately 43% more c-Fos cells than the other groups (each pairwise comparison P < 0.05; Fig. 6a). Amphetamine had an opposite effect on the number of c-Fos positive cells in the granular layer of cerebellar vermis lobules VI and VII in High-Active versus Control mice. This was indiciated by a significant interaction between line and amphetamine (F_1, 28_ = 4.15; P = 0.043). In Control mice, low-dose amphetamine increased c-Fos whereas in High-Active mice it decreased c-Fos, as indicated in Fig. 6b. Main effects of line and amphetamine were not significant, nor were any posthoc tests.

### Experiment 3: Adolescent males

#### Hits and False Alarms

**Figure 7:**
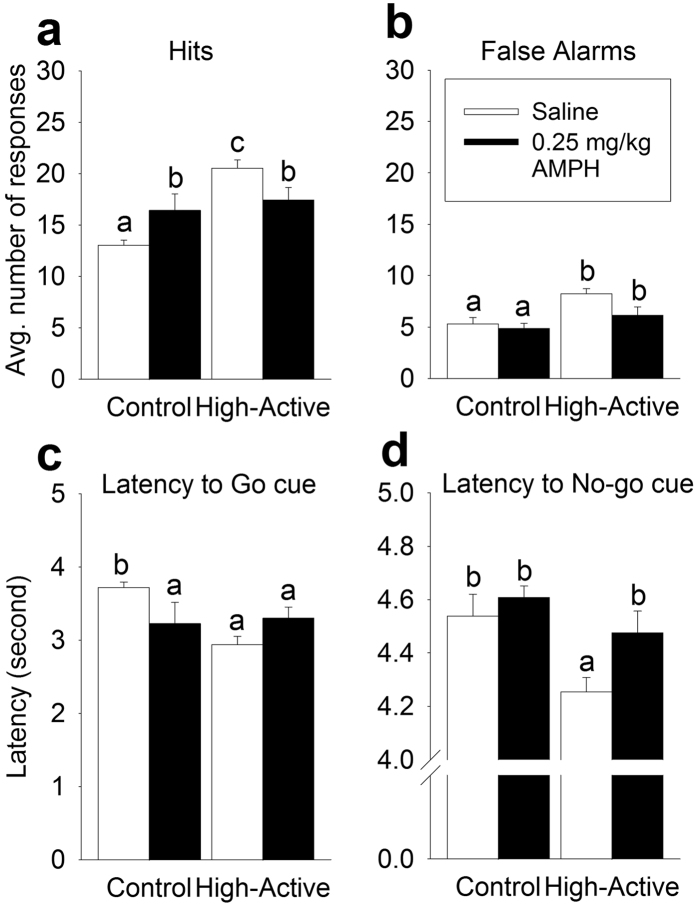
Experiment 3: Adolescent male response to low-dose amphetamine in Go/No-go testing. White bars indicate saline-exposed mice while dark bars represent 0.25 mg/kg amphetamine-exposed mice. Intraperitoneal injections were administered 15 minutes before performing Go/No-go testing. The sample sizes for the data shown are as follows: for the High-Active cohort, n = 5 received saline and n = 7 received 0.25 mg/kg amphetamine; for the Control cohort, n = 4 received saline and n = 5 received 0.25 mg/kg amphetamine. Bars with a different letters are significantly different from each other (P < 0.05). All data reflect the 10 day average across Go/No-go testing. (**a**) The average number of hits (correct nose poke in response to a light) (±SEM) in response to i.p. injections of either saline or 0.25 mg/kg d-amphetamine. (**b**) The average number of false alarms (incorrect nose pokes in response to a tone) (±SEM) in response to i.p. injections of either saline or 0.25 mg/kg amphetamine. (**c**) The average latency in seconds (±SEM) to respond to the presentation of a Go cue (light) when exposed to saline or 0.25 mg/kg amphetamine. (**d**) The average latency in seconds (±SEM) to (incorrectly) respond to a No-go cue (tone) when exposed to saline or 0.25 mg/kg amphetamine.

As ADHD presents during childhood/adolescence, the characterization of an adolescent cohort is critical to validate the High-Active model of ADHD. We hypothesize that High-Active male mice will exhibit impulsivity-related deficits within this developmental time period, further confirming face validity for the High-Active model. Therefore motor impulsivity and amphetamine response testing was conducted in adolescent High-Active males at a clinically relevant age, and in the preferentially affected sex^36^. The low dose of 0.25 mg/kg d-amphetamine had opposite effects on hits in adolescent High-Active mice as compared to adolescent Controls (Fig. 7a). This was indicated by a significant main effect of line (F_1,17_ = 11.9; P = 0.003), as overall High-Active mice made more hits than Controls, no main effect of treatment (saline versus amphetamine), and a significant interaction between line and treatment (F_1,17_ = 7.01; P = 0.017). The interaction was caused by amphetamine decreasing hits in High-Active while increasing hits in Controls. Hits, and the ability of amphetamine to attenuate High-Active hits is likely a correlated response to selection rather than a result of genetic drift (Table 1).

Adolescent High-Active males also displayed significantly greater number of false alarms than Controls (F_1, 17_ = 8.80; P = 0.009; Fig. 7b). Amphetamine had no significant effect in either line. Increased false alarms in the High-Active line are likely a correlated response to selection rather than genetic drift (Table 1).

#### Analysis of Covariance of False Alarms

Considering only mice exposed to saline, neither number of hits or home cage activity were significant predictors of false alarms. After correcting for number of hits, High-Active mice performed approximately 7 more false alarms relative to Controls (F_1,6_ = 10.8; P = 0.017). After correcting for home cage activity they displayed approximately 4 more (F_1,6_ = 9.5; P = 0.02). After correcting for both, they displayed approximately 9 more false alarms than Controls (F_1,5_ = 15.8; P = 0.011). These results indicate that the increased number of false alarms displayed by High-Active adolescent males is not a trivial consequence of their increased number of hits or hyperactivity.

#### Latency to Nose Poke

Amphetamine had an opposite effect on latency to respond to the Go cue in High-Active mice as compared to Controls (Fig. 7c). In High-Active mice, amphetamine increased latency to respond, whereas in Control mice it decreased latency. This was indicated by a significant interaction between treatment and line (F_1,17_ = 5.2; P = 0.035) and no significant main effects. Latency to respond to the No-go cue, i.e. false alarms, were shorter in High-Active mice than Controls but amphetamine had no significant effect (Fig. 7d). This was indicated by a significant main effect of line (F_1,17_ = 9.3; P = 0.010) but no effect of amphetamine or interaction between amphetamine and line. Differential responses to amphetamine in High-Active versus Control lines survived the test for genetic drift for hit latencies but not false alarm latencies (Table 1).

## Discussion

The main finding of the study is that 16 generations of selection for increased physical activity in the home cage results in increased motor impulsivity in the High-Active line relative to the Control line, suggesting that hyperactivity and impulsivity are inevitably entangled traits influenced by similar suites of genes in our lines (Table 1). This result was confirmed using two different versions of the Go/No-go test of motor impulsivity (Experiment 1) in adults and adolescents (Experiments 2 and 3), and in both males and females. Supporting the predictive validity of the High-Active model is the finding that amphetamine, widely used as a therapeutic for ADHD, ameliorates not only home cage hyperactivity^23^ but is also effective in reducing impulsivity-related traits. Moreover, High-Active mice displayed no impairment on the Y-maze spontaneous alternation task of attention, and demonstrated significant impairment in the accelerating rotarod task, which requires motor coordination, motor learning, and intact cerebellar function^35^. High-Active mice also displayed altered cellular activation in the cerebellar vermis and PFC in response to low therapeutic amphetamine administration, consistent with human literature implicating altered functionality in these regions associated with ADHD^37^. We conclude that the High-Active line, when used in conjunction with the concurrently bred Control line, represents a valuable model for exploring the etiology of the Hyperactive-Impulsive subtype of ADHD, and as a platform for new medication development.

The High-Active line demonstrates that hyperactivity and impulsivity are genetically entangled traits. Excessive motor impulsivity in the High-Active line was demonstrated in two separate assays, across distinct sensory cues employed to elicit behavioral inhibition^27^,^28^. In both cases, a central measure of impulsivity (false alarms) suggest High-Active impulsivity is unlikely due to genetic drift, and is more likely a correlated response to selection for hyperactivity, as reflected in Table 1. False alarms, or the mistaken “go” response to a No-go cue, indicate an impaired ability to withhold a prepotent, impulsive motor response in order to obtain a reward^27^. Furthermore, the duration of that withholding behavior is shortened in High-Active mice relative to Controls, and this deficient control over withholding impulsive behavior is also a correlated response to selection for hyperactivity (Table 1). Hits, or correct responses to the Go cue, are commonly evaluated in the context of Go/No-go paradigms. It is important to emphasize that hits are not a measure of impulsivity; hits are simply the developed prepotent motor response, and reflect activity or vigilance of the mice to respond to cues in the operant chambers. It should be appreciated, however, that some variation in response latency exists among strains tested depending on the specific Go/No-go protocol employed. As compared to the observed 2–4 s response latencies of High-Active and Control mice (Figs 3e,f and 7c,d), previous studies using inbred strains have demonstrated shorter latencies (under 1 s)^28^ while other studies have shown multiple strains respond within 2-3s^27^,^38^. Therefore the High-Active and Control mice may be considered less vigilant compared to inbred strains used in alternative Go/No-go protocols, raising the possibility that pure response inhibition alone is not reflected by these latency data. However, in the context of false alarms, hit measurements enables evaluation of the degree to which mice discriminate between Go and No-go cues. In Version 1, there is a clear differential response to a Go versus a No-go cue (Fig. 3c), while Version 2 presents a more challenging task wherein go and no-go cues elicit similar levels of responding (Fig. 3d). The latency of the mice to withhold the impulsive nose poke also underscores the discriminatory capacity of the cues; mice clearly inhibit responding to No-go cues in Version 1 (Fig. 3c), and if a mistake is committed, it occurs after a longer period of maintained inhibition as compared to a correct hit (Fig. 3e). While both versions of the Go/No-go task ultimately indicated higher measures of impulsive false alarms in High-Active mice, Version 1 cues elicited clearly discriminatory behaviors, and therefore allow us to more clearly parse out capacity for impulse control. Furthermore, Version 1 adheres to parameters established for 15 strains of mice in Go/No-go testing^27^ and is therefore ideal for this highly heterogeneous population. Version 1 was subsequently utilized in assessing motor impulsivity in our most extreme hyperactive population (Experiment 2, adult females; Fig. 5) as well as a translationally relevant cohort (Experiment 3, adolescent males; Fig. 7). In all cohorts, the High-Active line demonstrated increased motor impulsivity (false alarms) relative to Controls.

Although these data clearly establish that the hyperactivity and impulsivity measures collected are genetically correlated traits in our lines, the specific genes that relate to both traits are not known, nor are the mechanisms for how those genes exert their influence on hyperactivity and impulsivity understood. One possibility is that the association between impulsivity and hyperactivity is a trivial consequence of the method used to assess motor impulsivity; there may exist a linear relationship in which hyperactivity directly translates into excessive cue poking, and therefore increases non-specific operant responding (as seen in Fig. 5d). Indeed, previous rodent studies have found positive correlations between open field activity and impulsive lever pressing across 6 strains of mice^39^, and further found significant correlations between open field activity and impulsive escape behavior^40^. Moreover, the SHR model of ADHD has demonstrated that reducing hyperactivity via d-amphetamine administration also reduces impulsive lever pressing^41^. If there existed an established strategy to measure motor impulsivity independent of locomotor activity, then the trivial hypothesis would predict High-Active mice would not display impulsive motor behavior.

The other possibility is that certain genes are common to both hyperactivity and impulsivity, so the genetic changes that cause hyperactivity inevitably deteriorate impulse control as well. This “common genes” hypothesis of entangled impulsivity and hyperactivity is embraced in clinical literature; many heritability studies only seek to parse out the genetic constructs underlying the “Hyperactive-Impulsive” subtype from the “Inattentive” subtype^42^,^43^,^44^. Clinical diagnostic categories confirm the validity of this approach; there exists no separate diagnoses for “Hyperactive” versus “Impulsive” subtypes of ADHD in the DSM-V^45^. This hypothesis posits that the relation between impulsivity and hyperactivity is not trivially dependent on locomotion, and if there were a reliable method for testing motor impulsivity independent of locomotion, then High-Active mice would still display increased motor impulsivity relative to Controls. Our results from the analyses of covariance provide important evidence in favor of the common genes hypothesis by showing that the higher number of false alarms in High-Active mice relative to Controls is not completely explained by their high level of activity or tendency to respond in the operant chamber.

Multiple clinical and animal model studies corroborate the present High-Active impulsivity data to support the hypothesis that impulsivity and hyperactivity are distinct yet related constructs. The identification of specific loci underlying ADHD-like behavior using a recombinant inbred strain approach has been successful in advancing our understanding of its genetic basis. Previous work by Loos and colleagues have successfully utilized this approach to identify substrates of impulsive action and attention^46^,^47^. Furthermore, a study conducted by Wilkinson and colleagues demonstrates that genetic hyperactivity and impulsivity are indeed not trivially locomotor-dependent in mice^48^. Multiple inbred lines of mice displayed co-varying levels of activity and impulsive choice on a delayed-reinforcement paradigm, yet this phenomenon was not attributed to generalized activity, largely due to the absence of systemic effects within motor performance^48^. Another study demonstrates that activity levels (exploration in novel open field) and impulsivity (precue responding in the five-choice serial reaction time task) are not correlated and further purports that these traits may be mediated by disparate genetic influences, based on the systemic analysis of 12 different inbred mouse strains^49^. Pharmacological evidence has also implicated differential bases for motor impulsivity and hyperactivity; different doses of amphetamine uniformly increases locomotor activity while producing a complex dose response pattern on impulsivity in young male mice, indicating distinct neural mechanisms mediate hyperactivity and impulsivity^50^. We build on such previous work by evaluating behavioral impulsivity in the context of a highly genetically variable model of hyperactivity that may better approximate human variation.

The significance of the translational potential of the High-Active line in modeling ADHD is validated by evidence that low-dose amphetamine reversed hyperactivity and impulsivity-related traits, both core symptoms of ADHD. Amphetamine reduced false alarms (Fig. 5b), precue responding (Fig. 5c), random cue-side poking (Fig. 5d), and improved efficiency (Fig. 5e) in High-Active mice. The capacity of amphetamine to reduce so many facets of impulsive responding highlights the strong predictive validity of the model. Particularly striking are the efficiency data (Fig. 5e), which demonstrate dramatic evidence that amphetamine improves High-Active impulse control while simultaneously worsening Control impulse control. As would be expected from clinical and animal literature on the differential effects of psychostimulants^37^,^51^, Control mice either remained minimally affected by amphetamine in these domains of motor impulsivity (Figs 5c,d and 7b,d), or demonstrated a worsening of traits related to impulse control (Fig. 5b,e), indicating low-dose amphetamine modulates conditioned motor behavior in both lines to a common, middle ground. These results demonstrating that amphetamine ameliorates impulsive motor behaviors in the High-Active mice, while having either no effect or a detrimental effect in Controls, is consistent with a large literature on the rate-dependent effects of psychostimulant drugs^52^,^53^,^54^. This literature establishes that across multiple strains and individuals, those subjects that display high baseline responses, e.g., hyperactivity, impulsivity, will tend to display reductions in these responses from amphetamine, while hypoactive or marginally active subjects with appropriate impulse control will tend to increase these behaviors in response to amphetamine^54^,^55^. It should be noted, however, that an inevitable consequence of measuring impulsivity and amphetamine response in a translationally-relevant adolescent cohort necessitated social isolation and mild food restriction at an earlier age than usual (PND 40 versus 60). Therefore it is possible that these factors contributed to the differential response between lines or to amphetamine. Taken together with adult female data which were isolated at a usual age (PND 60), results strongly suggest amphetamine has a specific capacity to ameliorate impulsive behavior in High-Active mice. Moreover, the paradoxical result of amphetamines between the lines is consistent with a large empirical literature on the rate dependent effects of amphetamine in rodents.

Unequal neural activation within the prelimbic and infralimbic cortices (PFC) in response to low-dose amphetamine provides further construct validity for this model of genetic hyperactivity-impulsivity. The PFC is consistently identified as a region of critical dysfunction in the etiology of ADHD; lesioning the PFC recapitulates the hyperactivity, impulsivity, and inattention of ADHD^56^,^57^,^58^, and hypofrontality, or weakened PFC functioning, is commonly identified in ADHD imaging studies^24^,^25^. We therefore hypothesized that the neuronal activation of the PFC would be reduced in the High-Active mice and that amphetamine would increase activation, but the data indicate this relationship is more complex than we previously thought. Activation of the PFC was reduced in High-Active mice relative to Controls, but this effect was driven by the lack of response of the High-Active mice to amphetamine (Fig. 6a). Altered functionality or expression of amphetamine targets (dopamine or norepinephrine transporters) in the High-Active PFC is a likely explanation for the minimal response to amphetamine^10^,^14^. The pattern of c-Fos expression shown in Fig. 6a bears a strikingly similarity to the pattern of c-Fos induced by methylphenidate in the frontal association and orbitofrontal cortices of the dopamine transporter knockout (DAT-KO) mouse model of ADHD. In the control (wildtype) mice, methylphenidate drastically increased c-Fos expression whereas in the DAT-KO, the stimulant had no effect^59^. This remarkable consistency speaks to the potential of the High-Active line to provide converging evidence regarding the genetic nature of ADHD when used in conjunction with other models.

Differential regulation of the cerebellum by low-dose amphetamine in the High-Active and Control lines further supports the validity of the High-Active model of ADHD. The dysregulation of the cerebellar vermis has become broadly accepted a potential etiological component of ADHD in clinical imaging studies^16^,^60^. Firstly, in an effort to assess the gross cerebellar functionality of our High-Active and Control lines, we employed a cerebellar-dependent accelerating rotarod task^35^. As hypothesized, the High-Active mice demonstrate deficits in the motor coordination required to successfully manage the task (Fig. 4c,d), suggesting underlying neural dysfunction of the region. Therefore, post-mortem immunohistochemical analysis of the activation of the granular layer of posterior vermal lobules VI and VII in response to saline or 0.25 mg/kg amphetamine was undertaken to determine whether hypo-activation correlated with this poor rotarod performance. Contrary to our hypothesis, High-Active mice showed increased activation of this region relative to Controls, an effect which is reversed by exposure to amphetamine (Fig. 6b). Previous work has confirmed the projection of dopaminergic ventral tegmental area fibers onto the granule layer of the cerebellar cortex^61^; therefore it is possible that aberrant baseline dopamine signaling increased baseline activation of vermal lobules VI and VII in the High-Active line, which is corrected by the action of amphetamine on neural substrates that project to vermal granular layer. The increased c-Fos expression in Controls exposed to amphetamine is expected, based on previous studies which have demonstrated acute d-amphetamine increases c-Fos expression in posterior lobules in rodents^62^. Taken together, the paradoxical effect of amphetamine on vermal activation implicates dopaminergic and noradrenergic dysfunction of upstream projections in the High-Active line.

The majority of these data consistently indicate that the High-Active line of mice is suitable for the study of Hyperactive-Impulsive ADHD. However there are several caveats that should be appreciated when evaluating and interpreting these findings. Firstly, operant training resulted in high attrition rates within Control saline-exposed mice in Experiments 2 and 3 due to failure to acquire nose-poking behavior. The high attrition rate indicates that Control mice demonstrate wide variation in behavior, and some may have difficulty learning the task and/or are unmotivated at baseline. Excluding such mice ensures a fair comparison between Control and High-Active mice, i.e. comparisons are only made between cohorts which show similar levels of learning and motivation. Furthermore, in comparing our attrition rates to the attrition rates found across 15 strains^27^, it becomes clear that some strains demonstrate more difficulty in completing the training phases than others. In particular, one strain (NZB/B1NJ mice) had 10 mice excluded for failure to complete training. The NZB strain is closely related to the NZO strain which was used in the founder CC population^22^ for the High-Active and Control lines, therefore variation in attrition rates is expected.

Ultimately, the High-Active line represents a useful model for exploring the etiology of the “predominantly Hyperactive-Impulsive” subtype of ADHD. High-Active mice demonstrate significant home cage hyperactivity and motor impulsivity-related traits, yet do not demonstrate significant inattention relative to Controls, as evidenced by spontaneous alternation behavior in the Y-maze (Fig. 4b) and vigilance for hits in the Go/No-go task (Fig. 3c,d). Certainly no rodent model perfectly manifests the multifaceted presentation of ADHD, but we propose the High-Active line demonstrates evidence of face, construct, and predictive validity which makes it suitable for testing hypotheses regarding the Hyperactive-Impulsive subtype of ADHD. Selective breeding has generated a heterogeneous population reflective of human genetic complexity, and in this way provides the truly unique opportunity to 1) evaluate whether candidate genes currently associated with ADHD are also correlated with hyperactivity-impulsivity in the High-Active model, and/or 2) uncover novel gene pathways underlying hyperactive-impulsive behavior. The identification of such novel genes may provide new targets for candidate gene studies in clinical populations. Furthermore, the specific behavioral and pharmacological contributions of novel genes may be elucidated using powerful transgenic technologies available for the mouse^63^. The High-Active model has also been validated with low-dose amphetamine, and is therefore suitable for testing novel compounds to ameliorate ADHD. Lastly, the concurrent breeding of the Control and High-Active lines from the same Collaborative Cross founder population allows for powerful, statistically valid comparisons of any behavior, drug response, or genetic factors which may differ between the two lines. We conclude that the High-Active line should be aggressively used to elucidate genetic and neurological underpinnings of ADHD-related disorders and as a platform for medication development.

## Additional Information

**How to cite this article**: Majdak, P. *et al*. A new mouse model of ADHD for medication development. *Sci. Rep.* **6**, 39472; doi: 10.1038/srep39472 (2016).

**Publisher's note:** Springer Nature remains neutral with regard to jurisdictional claims in published maps and institutional affiliations.

## Supplementary Material

Supplementary Information

## Figures and Tables

**Table 1:** Evaluation of secondary traits as correlated responses to selection for home cage hyperactivity.

Trait	Expt	Figure	High-Active line	Control line	p-value	*h*^*2*^	F	n	95% CI	D_y_
Home Cage Activity	1	2a	0.92 (±0.10)	0.44 (±0.06)	0.0004	0.33	0.06217	12	1.26	**1.54**
Hits	1	3c&d	28.2 (±0.44)	23.7 (±1.37)	0.007	0.4	0.06217	12	1.29	**1.30**
Hits latency	1	3e&f	1.53 (±0.12)	2.24 (±0.22)	0.007	0.18	0.06217	12	1.21	−1.17
FA (V1)	1.1	3c	16.5 (±1.39)	9.59 (±1.50)	0.005	0.17	0.06217	12	1.20	**1.82**
FA (V2)	1.2	3d	28.3 (±0.38)	24.8 (±1.40)	0.020	0.17	0.06217	11	1.25	**1.63**
FA latency (V1)	1.1	3e	3.43 (±0.14)	4.16 (±0.12)	0.002	0.06	0.06217	12	1.16	−**2.06**
FA latency (V2)	1.2	3 f	1.39 (±0.09)	1.91 (±0.26)	0.053	0.06	0.06217	11	1.21	−**1.27**
Precue response	1	—	19.15 (±3.08)	18.14 (±2.7)	NS	—	—	—	—	—
Cue side poke	1	—	142.7 (±17.2)	156.6 (±33.9)	NS	—	—	—	—	—
Efficiency	1	—	18.60 (±2.5)	18.40 (±2.26)	NS	—	—	—	—	—
Home Cage Activity	2	2b	1.36 (±0.25)	0.53 (±0.11)	0.013	0.33	0.06217	13	1.22	0.97
Y-maze arm entries	2	4a	63.8 (±4.18)	42.0 (±3.12)	0.0001	0.39	0.06217	18	1.11	**1.30**
Rotarod latency (s)	2	4c	31.7 (±2.06)	43.3 (±3.90)	0.015	0.44	0.06217	18	1.13	−0.77
Hits	2	5a	19.8 (±2.10)	12.6 (±3.47)	0.049	0.4	0.06217	13	1.25	1.15
AMPH on Hits	2	5a	0.43 (±1.28)	6.42 (±1.77)	0.013	0.21	0.06217	13	1.18	−**2.80**
Hits latency	2	—	1.56 (±0.1)	1.82 (±0.19)	NS	—	—	—	—	—
AMPH on Hits latency	2	—	−0.14 (±0.09)	−0.23 (±0.10)	NS	—	—	—	—	—
FA	2	5b	9.45 (±1.59)	3.20 (±0.78)	0.007	0.17	0.06217	13	1.16	**1.62**
AMPH on FA	2	5b	−1.35 (±1.1)	3.87 (±0.51)	0.001	0.21	0.06217	13	1.18	−0.68
FA latency	2	—	2.11 (±0.07)	2.36 (±0.03)	NS	—	—	—	—	—
AMPH on FA latency	2	—	0.03 (±0.05)	−0.13 (±0.03)	0.015	0.21	0.0622	13	1.18	1.17
Precue response	2	—	19.96 (±4.8)	4.3 (±0.1.54)	0.002	—	—	—	—	—
Cue side poke	2	—	83.28 (±23.2)	23.1 (±6.49)	0.0008	—	—	—	—	—
Efficiency	2	—	25.53 (±4.5)	73.6 (±16.4)	0.005	—	—	—	—	—
Home Cage Activity	3	2c	0.72 (±0.11)	0.31 (±0.04)	0.006	0.33	0.06217	11	1.31	**1.38**
Hits	3	7a	20.5 (±0.82)	13.0 (±0.50)	0.003	0.4	0.06217	11	1.33	**4.92**
AMPH on Hits	3	7a	−3.10 (±1.2)	3.42 (±1.59)	0.008	0.21	0.06217	11	1.26	−**1.93**
Hits latency	3	7c	2.94 (±0.12)	3.72 (±0.08)	0.001	0.18	0.06217	11	1.25	−**3.53**
AMPH on Hits latency	3	7c	0.36 (±0.15)	−0.49 (±0.3)	0.017	0.21	0.06217	11	1.26	**1.66**
FA	3	7b	8.22 (±0.50)	5.30 (±0.64)	0.009	0.17	0.06217	11	1.25	**2.44**
AMPH on FA	3	7b	−2.08 (±0.8)	−0.46 (±0.5)	NS	—	—	—	—	—
FA latency	3	7d	4.25 (±0.05)	4.53 (±0.08)	0.010	0.06	0.06217	11	1.21	−**2.03**
AMPH on FA latency	3	7d	0.22 (±0.08)	0.07 (±0.04)	NS	—	—	—	—	—
Precue response	3	—	13.3 (±0.89)	10.2 (±1.2)	NS	—	—	—	—	—
Cue side poke	3	—	60.9 (±9.27)	51.1 (±15.8)	NS	—	—	—	—	—
Efficiency	3	—	20.9 (±1.45)	24.6 (±2.7)	NS	—	—	—	—	—

Mean values (±SEM) for each line (“Control” and “High-Active”) are shown along with the p-value resulting from the t-test comparing the line means. In cases where published heritability estimates were available, further statistics to evaluate the likelihood that the line differences were attributable to selective breeding for hyperactivity, as opposed to genetic drift or founding effects are provided as follows the work of Henderson^64^,^65^ and Konarzewski^32^. “FA” denotes false alarms for Version 1 (V1) or Version 2 (V2); “AMPH on” refers to the influence of 0.25 mg/kg d-amphetamine administered via i.p. injection on a trait calculated by subtracting the average saline response from each individual amphetamine response. “P-value” refers to the t-test comparing the High-Active to Control line. Heritability (“*h*^2^”) estimates were available for analyses of home cage activity^26^, operant measures (hits, false alarms, hits or “go” latency, false alarms or “no-go” latency)^27^, Y-maze arm entries^66^, and an average of the three days of rotarod^67^,^68^. “Hits” and “Hits latency” data reflected in the Experiment 1 cohort are collapsed across Versions 1 and 2, as the Go signal was identical in both. “F” refers to the inbreeding coefficient estimated from the pedigree. “n” refers to the number of families represented in the analyzed trait. “95% CI” refers to the 95% confidence interval for D_y_ expected by genetic drift^23^. “D_y_” refers to the difference between lines in standardized phenotypic SD units. D_y_ absolute values exceeding the 95% CI are shown in bold, and provide evidence that the secondary trait has evolved as a correlated response to selection for hyperactivity in the home cage. If heritability estimates were not available in the literature, or if line differences were not significant by t-test, then the traits were not further analyzed for genetic drift (as indicated by dashes).
