# Supplementary material for: A new mouse model of ADHD for medication development
Source: Sci Rep. 2016 Dec 20;6:39472. doi: 10.1038/srep39472 (PMC5171883; doi:10.1038/srep39472)
Supplement: Supplementary Information [file srep39472-s1.doc]

**Supplementary Information**

A new mouse model of ADHD for medication development

Petra Majdak^1,2^, John R. Ossyra^3^, Jessica M. Ossyra^4^, Adam J. Cobert^5^, Gabrielle C. Hofmann^6^, Stephen Tse^2^, Brent Panozzo^2^, Elizabeth L. Grogan^2^, Anastassia Sorokina^2^, and Justin S. Rhodes^1,2,7^.

^1^The Neuroscience Program, University of Illinois

^2^The Beckman Institute for Advanced Science and Technology, University of Illinois

^3^Oak Ridge National Laboratory, University of Tennessee, Knoxville

^4^College of Engineering, University of Tennessee, Knoxville

^5^Department of Food Science and Technology, University of California, Davis

^6^College of Veterinary Medicine, University of Illinois

^7^Department of Psychology, University of Illinois

**Supplementary Methods**

**Operant Training and Go/No-go testing**

Training for all experiments proceeded identically, and was modeled after a paradigm established by Suzanne Mitchell and colleagues^26^. Each mouse was randomly assigned to receive a cue light in either the right or the left nose poke hole for the duration of the training and testing; mice were counterbalanced for side within each line and dose. Mice demonstrated no differences in behaviors due to the side on which the cue light was illuminated (data not shown). Training Phases 1 and 2, and Go/No-go testing were run during the dark cycle (which lasted 8:00 AM until 8:00 PM) 7 days a week until completion of the testing for all mice. Mice in Experiments 1 and 2 were continually food restricted to 90% of their free feeding weight, while mice in Experiment 3 had food removed from their home cage 3 hours prior to operant training/testing, after which time ad libitum feeding resumed.

*Training Phase 1*

During a Phase 1 training session, the mouse is placed in the chamber, and experiences 60 Go cue presentations (nose poke recess light) over the course of approximately 1.5 hours (varies depending on mouse performance). Firstly, the house light is illuminated for a variable fore period of 9-24 seconds (which ensures the mouse cannot predict cue presentation), after which time the nose poke recess light is illuminated (the “Go” cue) for 30 seconds. A nose poke in the illuminated hole will be reinforced for via the presentation of 0.01 cc of 10% sucrose solution in the center nose poke recess for 3 seconds. This behavior is considered a hit. A darkened intertrial interval of 10 seconds follows reward delivery, and a new trial begins. Typically, there are two alternative situations may arise within training, namely 1) the mouse does not nose poke during the Go cue, in which case no reward is delivered and the trial resets with the darkened intertribal interval, and 2) the mouse pokes prematurely in the cue hole (a precue response, defined as 3 seconds prior to the presentation of the Go cue), and the mouse is immediately penalized via a trial reset, to avoid inadvertently collecting response data that is non-specific (combining precue with cue responding). Once a mouse successfully completes 30 correct nose pokes in response to the Go cue within 40 min for two consecutive sessions (days), the animal moves on to Phase 2.

*Training Phase 2*

Phase 2 has an identical structure to Phase 1, except that the cue is illuminated for only 10 seconds rather than 30 seconds. Similarly, once a mouse successfully completes 30 correct nose pokes in response to the Go cue within 40 min for two consecutive sessions, the animal moves on to Go/No-go testing.

*Go/No-go Testing*

After successfully completing training Phases 1 and 2, Go/No-go testing is conducted over daily sessions. Each daily Go/No-go session lasted for either 60 trials or until 1 hour has passed. During this time, there are 30 presentations of a Go cue (nose poke to cue light) randomly interspersed with 30 presentations of a No-go cue (tone in Version 1, concurrent tone and light in Version 2). The duration of either cue is 5 seconds. A nose poke during the presentation of a Go cue (hit) results in the expected 10% sucrose solution reward. Additionally, withholding a nose poke response during the presentation of the No-go cue (a “correct rejection”) also results in the 10% sucrose reward. A mistaken nose poke impulse during the presentation of the No-go cue (a “false alarm”) immediately results in a darkened intertrial interval and no reward.

**Supplementary methods for Experiment 1: Evaluating two discrepant Go/No-go procedures**

In Experiment 1, two established versions of the Go/No-go task were assessed. Version 1 utilizes a tone for the No-go cue^26^, while Version 2 utilizes a concurrent tone and light for the No-go cue^27,28^. Clearly, Version 2 is more challenging for the mice, as they have been trained previously to nose poke in response to a light only, but now the mice must withhold nose poking behavior only when the tone is played concurrently with the illumination of the light. Therefore, to accommodate the increased difficulty of this task, mice underwent 20 days of Go/No-go testing in Version 2 while mice underwent only 10 days of Go/No-go testing in Version 1. The additional 10 days of testing was added to Version 2 in order to allow additional time for the mice to acquire this difficult rule (nose poke for cue light, but withhold for cue light plus tone). However, analysis of key variables such as hits, false alarms, and latency in Version 2 of did not differ when averaged across the first 10 days of testing versus the full 20 days of testing (data not shown). This indicates the mice were not able to fully acquire the rules of the more difficult Version 2, as their performance did not improve despite being given twice as long to acquire the contingencies and maximize reward opportunities.

**Criteria for excluding mice**

Mice that did not pass the training phases (i.e., did not acquire the nose poke behavior) were excluded (Experiment 2: n=6 Controls receiving saline; Experiment 3: n=4 Controls receiving saline, and n=2 Controls receiving 0.25 mg/kg amphetamine). Mice which completed training and testing on Go/No-go but were not collecting the delivered sucrose reward for their nose poke (collecting less than 40% of the time) were not included in the final analysis (Experiment 1 Version 1: 2 High-Active males, 1 High-Active female, and 1 Control female were excluded; Experiment 1 Version 2: 1 High-Active male, 2 High-Active female, 2 Control males and 1 Control female; Experiment 2: n=4 Controls receiving 0.25 mg/kg amphetamine, n=2 High-Actives receiving saline; Experiment 3: n=2 Controls receiving saline, n=2 Controls receiving 0.25 mg/kg amphetamine, n=5 High-Actives receiving saline, and n=3 High-Actives receiving 0.25 mg/kg amphetamine). These mice were excluded due to the fact their unknown motivational influence cannot be accounted for when analyzing behavioral performance. The final sample sizes are shown in the manuscript proper.
